# Multifractal analysis reveals music-like dynamic structure in songbird rhythms

**DOI:** 10.1038/s41598-018-22933-2

**Published:** 2018-03-15

**Authors:** Tina C. Roeske, Damian Kelty-Stephen, Sebastian Wallot

**Affiliations:** 1 Max Planck Institute for Empirical Aesthetics, 60322 Frankfurt, Germany; 2 Department of Psychology, Grinnell College, Grinnell, 50112 IA USA

## Abstract

Music is thought to engage its listeners by driving feelings of surprise, tension, and relief through a dynamic mixture of predictable and unpredictable patterns, a property summarized here as “expressiveness”. Birdsong shares with music the goal to attract its listeners’ attention and might use similar strategies to achieve this. We here tested a thrush nightingale’s (*Luscinia luscinia*) *rhythm*, as represented by song amplitude envelope (containing information on note timing, duration, and intensity), for evidence of expressiveness. We used multifractal analysis, which is designed to detect in a signal dynamic fluctuations between predictable and unpredictable states on multiple timescales (e.g. notes, subphrases, songs). Results show that rhythm is strongly multifractal, indicating fluctuations between predictable and unpredictable patterns. Moreover, comparing original songs with re-synthesized songs that lack all subtle deviations from the “standard” note envelopes, we find that deviations in note intensity and duration significantly contributed to multifractality. This suggests that birdsong is more dynamic due to subtle note timing patterns, often similar to musical operations like *accelerando* or *crescendo*. While different sources of these dynamics are conceivable, this study shows that multi-timescale rhythm fluctuations can be detected in birdsong, paving the path to studying mechanisms and function behind such patterns.

## Introduction

### Structural analyses of birdsong

Birdsongs are complex vocal sequences unfolding with rich rhythmic and melodic structure across time. What determines the dynamic structure of a species’ song? Many structural analyses of song have focused on how acoustic characteristics of song represent *adaptations* to ecological conditions, for instance how a habitat’s specific sound propagation/attenuation properties affect song structure^1–11^, or how structural aspects are affected by simultaneous singing of other hetero- or conspecific singers^6,12–15^ or the energetic cost of producing different sounds^16–19^. The structural aspects investigated were either high-level units like repertoires, song types, overall song/inter-song pause duration, or the occurrence of specific element categories such as trills, whistles, long, high-pitched, modulated notes^6,20^, or of discrete types of notes^13^.

Another line of research on birdsong structure has been concerned with the question what kind of algorithms the avian brain might use when generating note sequences, conceptualizing birdsong *syntactically*, i.e. as concatenations of distinguishable notes (“syllables”) whose transitions follow regular principles^21^. Analysis inspired by formal language theory has modeled different species’ syntax in terms of simple first-order sequence generation in which current states only depend on just-previous states^21,22^, or using slightly more complex sequence generation models that require a slightly longer memory^23^. (Note that birdsong research uses the term “syntax” in a very basic sense that encompasses any way of structuring a sequence of elements, even if the resulting structure is very simple. In contrast, linguistic research uses the term commonly with a strong focus on the complex, recursive structures found in human language).

Beyond the questions of ecological adaptation on the one hand and syntax generators on the other hand, we can take yet another perspective and focus on the proximate function of song to engage and attract a listener^24^. Which aspects of song structure could be specifically designed to attract and hold the attention of conspecific birds? As this is a function that has been suggested to be shared by birdsong and music^24,25^, research on human music might contribute relevant insight about what kinds of structures might be effective in fulfilling this goal.

Music psychology has put forth the hypothesis that what makes music attractive for listeners is its *dynamically fluctuating predictability*^26,27^: That is, by building and breaking expectations on multiple timescales, music is thought to create a dynamic succession of different feelings. Stereotypic/predictable patterns result in a sense of fulfillment, relief, and ease of processing; unexpected/variable patterns in surprise, and delay of an expected pattern in tension^27–29^. The constant fluctuation between variable/unpredictable and stereotyped/predictable patterns is believed to effectively attract and hold a listener’s attention.

**Figure 1:**
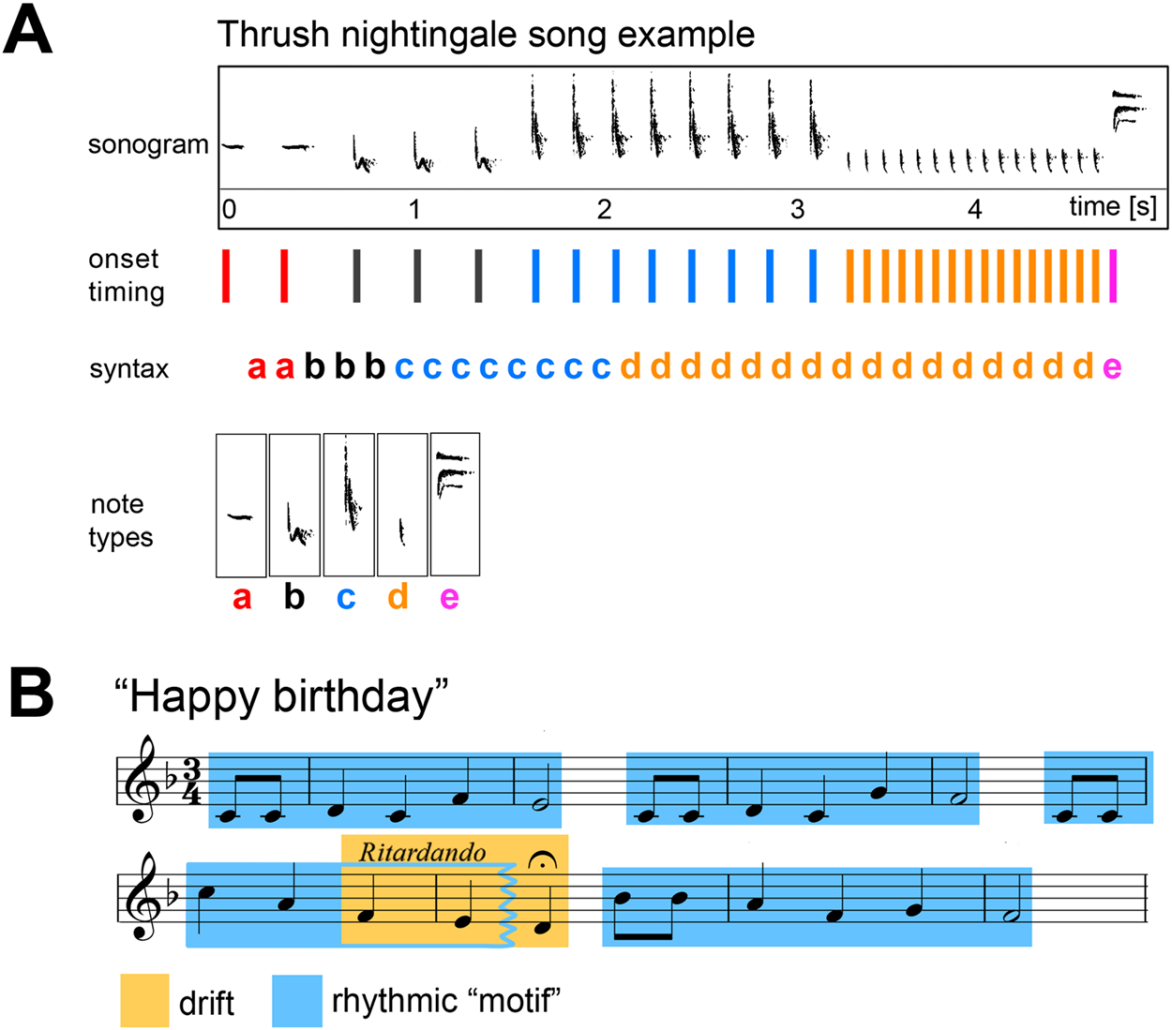
Examples illustrating rhythmic structure in music and birdsong. (**A**) Example sonogram of a thrush nightingale song consisting of five note types (a–e). The song’s rhythmic structure depends on (1) note intensity and duration (as apparent in the sonogram), (2) note syntax (i.e. sequential arrangement of notes of specific intensities and durations), and (3) timing of note onsets, which may either be under control of the bird, or an epiphenomenon of stringing together the vocal gestures required to produce the note sequence. (**B**) Score of the song “Happy birthday”, with blue shading indicating a recurring rhythmic motif: note timing in the different instances of the motif is the same, while pitch is variable. The third rendition of the motif is slightly modified at the end, containing one additional note instead of ending on a half note, which is here symbolized by the jagged right edge of the blue box. Yellow shading indicates a drift (slowing down of notes, or *ritardando* in musical terminology).

We suggest using the term *expressiveness* for such fluctuations that may be suited to engage a receiver’s attention^30,31^. The present study was motivated by the underlying question whether birds might make use of similar mechanisms to attract their listeners’ attention, i.e. whether birdsong features expressiveness based on balancing predictable and unpredictable patterns across multiple timescales. We set out to provide a first step to approach this question, by testing whether we can actually identify structures in birdsong that are suggestive of dynamically fluctuating predictability. With multifractal analysis, we adopted a method that has been used before to quantify multi-timescale fluctuations in signal variance (for details, see section “Multifractal analysis as a test for expressiveness”). Here, we use multifractal analysis to test a songbird’s *rhythm* for evidence of such dynamic structure. We use the term rhythm to describe the temporal patterning of the song, which includes timing of individual sounds, accents, and groupings^32^. The species investigated is the thrush nightingale, a songbird with a repertoire of 12–40 distinct song types per individual (a song type being a bout of continuous singing of about ~5–15 s, flanked by silence) sung in immediate variety, i.e. the same song type is not usually repeated directly^33^. Each song type contains about 4–10 different note types that have each a distinct spectral shape^34–36^; see Fig. 1A. Thrush nightingale song sounds highly “rhythmic” to human listeners^37,38^, perhaps because it contains very slow as well as very fast subphrases, and many repetitions.

### Rhythm as note timing in birdsong

For humans, the perception of a rhythm relies on two main components: (1) the timing of single notes, and (2) the timing of accents, i.e. timing of moments of relatively higher amplitude in the note sequence^32^.

Surprisingly little is known about songbirds’ rhythms^39^. Rhythm, as the *timing of individual notes within the sequence* has rarely been investigated. One of the few exceptions is a recent study showing that note timing in zebra finches is structured by an underlying fast, isochronous pulse that is very stable within individuals^40^, but relative timing of notes to each other has not been systematically investigated here. This also applies to a study by Saar *et al*.^41^, who developed a method to visualize how zebra finch motif length increases across development by adding more notes. Sasahara *et al*.^42^ have come up with an interesting visualization of the dynamics of rhythm development in Bengalese finch song. Their “rhythm landscape” visualization captures rhythm in terms of its most basic unit, onset-to-onset times, tracking their differentiation across development. Limiting the analysis to single note transitions, this approach does not address how rhythmic units are sequentially arranged in the song.

What could be the factors that are driving sequential note timing in the song of thrush nightingales? First, there’s the possibility that birds do not control note *timing* at all, but only the sequential arrangement of note types (syntax; see Fig. 1A). Variation in note timing could merely reflect random deviations or be a trivial consequence of note choice, while the process under control of the bird would be to generate a sequence of specific note types. In that case, our human impression that thrush nightingale song sounds “rhythmic” would reflect a human perceptual bias that might not be shared by the birds, which would conceptualize their songs as sequences of elements, irrespective of internal timing.

Alternatively, note timing could be under control of the birds. In this case, they would be able to use sound timing and/or accentuation to generate specific rhythms. They might then – like musicians – *make use* of note timing to achieve expressiveness, driving their listeners’ expectations mixing predictable and unpredictable timing patterns.

While these two scenarios – rhythm as controlled by the bird or not – will have a strong impact on temporal structure of the sequence, note that other influencers may exist: Certain gradual changes in timing/amplitude patterns on the relevant time scales (of up to about 1 s) might arise from fluctuations in attention and possibly arousal, or from physical constraints on breathing or sustained muscle activity. Our study aims at identifying and quantifying dynamic fluctuations in predictability, whereas future research is needed to identify their sources.

### Expressiveness in the rhythm of music and birdsong

What does it mean that a rhythm is expressive, in the sense that its predictability/variability is fluctuating? Two strategies commonly used in music to generate such expressiveness are *recurrent patterns* and *drifts*. An example for a *recurrent pattern* in rhythm is a rhythmic motif recurring periodically within a musical sequence. The rhythm can be realized by different note types, timbres, etc., while note timing and accent within the motif are fixed. *Drifts* are successive increases or decreases in rhythmic features, like in an *accelerando* (accelerating notes) or *ritardando* (slowing down of notes). Figure 1B shows the musical score of the song “Happy birthday”, which contains examples for both a recurrent rhythmic motif and a drift.

Both recurrences and drifts unfold across a broad range of intermediate to long timescales above transitions of individual notes, and can thus determine predictability across (sub-)sequences of notes. For instance, during an *accelerando* phrase, the listener can predict the incoming notes to continue picking up speed, but also the acceleration to stop at some later point and lead over to a different kind of rhythm. Songbirds, like musicians, might use drifts or recurring rhythmic motifs to enable their listeners to form rhythm expectations, which can then be fulfilled, delayed, or broken.

**Figure 2:**
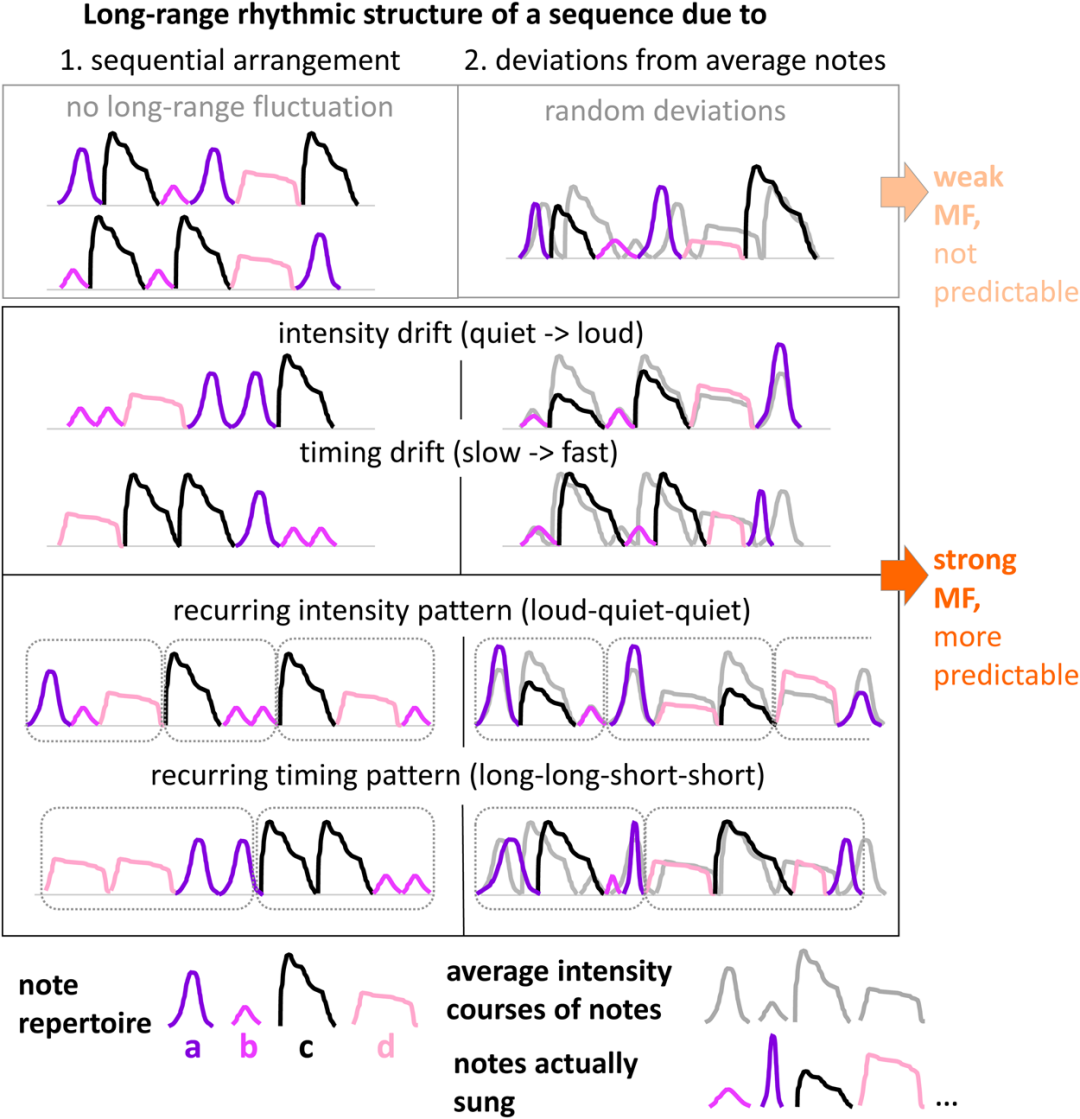
Impact of sequential arrangement vs. timing deviations on rhythm. Colored lines: amplitude envelopes of four notes types (a–d). Left: If sequential arrangement of notes is independent of note envelope characteristics, no systematic long-range structure emerges (top). Otherwise, arrangement may yield drifts or recurring patterns in intensity or timing (bottom). Right: Deviations of timing and amplitude from average rhythm may be random (top) or generate drift or recurring patterns (bottom). Multifractality (MF) values of drifting sequences (or those including recurring timing patterns) should exceed those without, and the additional structure added by drifts and recurrent patterns increases predictability of the sequence’s time structure.

In a thrush nightingale’s song, such expressive recurrence patterns and drifts could in principle be generated via two different mechanisms: (a) by *sequential arrangement* (syntax) of specific notes and pauses (Fig. 2, left), or (b) by adding to a given note sequence subtle *deviations* in individual note timing and/or amplitude (just like the musical operations of *accelerando*, *ritardando*, swinging rhythms in jazz^43^, or *notes inégales* [unequal notes in French Baroque^44^] would do). This way, note timing will slightly deviate from the “exact” rhythm as imposed by the sequential arrangement of note types (Fig. 2, right). The two mechanisms have different implications for the neural processes generating song and contributing to its timing (see Discussion for details).

Interestingly, music makes use of both mechanisms to generate long-range rhythmic structure, in a controlled and intentional way: Sequential arrangement (of notes of specific pitch and duration) has been shown to result in long-range correlations in the scores of Bach’s three-voice sinfonias^45^, and subtle deviations from “exact” rhythm during a musical performance show long-range correlations which listeners recognize and prefer over both exact rhythm or rhythm with *random* deviations^46,47^.

### Multifractal analysis as a test for expressiveness

Multifractal analysis is a method designed to test a signal for *fluctuations in variance across different time scales*. Variability is closely related to predictability (albeit in a non-trivial way that is heavily influenced by time-scale: adding random variability to a signal on short time-scales will decrease its predictability, while adding certain kinds of long-range variability like in a slow crescendo will increase predictability on shorter time-scales). Multifractal analysis is well suited to capture expressiveness, which we defined here as a mixture of predictable and unpredictable patterns across multiple timescales. In the following we will show that multifractal analysis can be successfully applied to detect such expressiveness in birdsong.

Variability fluctuations across different timescales can be described as *changes in long-range correlations*. They way multifractal analysis uncovers such changes in long-range correlations in a signal is by calculating standard deviation over time windows of many sizes: If these changes are not random (for instance, because a pattern recurs at different times in the signal, or a drift affects multiple consecutive time windows), they have multiple fractional exponents across time (and are called “multifractal”). The larger the range of these exponents, the more does variability over time reflect interactions across multiple timescales, as opposed to merely local relationships^48,49^. A birds’ rhythm that contains long-range structure, due to recurring rhythm patterns or drifts, would thus result in a wider multifractal range than note sequences lacking such features.

We first tested whether thrush nightingale rhythms exhibit such long-range correlations, by performing multifractal analysis on the songs’ amplitude envelopes (which contains information about both note timing and intensity/accentuation). Next, we tested for the role of sequential arrangement vs. timing/intensity deviations. To this end, we generated synthetic songs that lack all subtle deviations, but maintain original note order, to compare their multifractality to the original songs. To generate these “average-rhythm” songs, we averaged for each note type the amplitude envelope across all instances, and finally re-synthesized songs from these averaged envelopes using the original *order* of notes (see Methods). These average-rhythm songs are like a “mechanic” version of the original songs, with each note and note transition being completely stereotyped in timing and intensity. If the birds make use of timing/intensity deviations in a systematic way to increase expressiveness, the original amplitude envelopes should be more multifractal than their re-synthesized average-rhythm versions.

## Methods

### Data and processing

Song data consisted in 24 songs from a single 2:59-min field recording of one thrush nightingale, recorded on May 9, 2009, in the Biebrza Marshes in Poland by bird watcher Tomas Belka. We obtained the recording from the xeno-canto birdsong library (http://www.xeno-canto.org, recording #XC75409). Sampling rate of the recording was 48 kHz; no further information on the bird is available (e.g. we don’t know his age, or breeding status). We used “Sound Analysis for Matlab” (SAM, by Sigal Saar) to extract amplitude envelopes in 10 ms windows and 1 ms steps. Multifractal analysis was performed using the “Multifractal detrended fluctuation analyses” toolbox for Matlab from Espen Ihlen^48^. Inferential statistics were also performed in Matlab (Version: R2015b).

### Note segmentation and identification

Several seconds separate distinct thrush nightingale songs. Within songs, we identified note boundaries by taking the difference between two Hodrick-Prescott (HP) filterings of the amplitude envelope: (1) HP filter coefficient = 50; (2) HP filter coefficient = 5 * 10^7^) on the 1000 Hz amplitude envelope. We set between-note pauses to zero amplitude, since we would not want any variability of silent pauses to contribute to the measured multifractality. As a robustness check, we performed an alternate analysis of the zero-pause and the original envelopes in parallel, which showed that no significant difference in multifractality resulted from this cleaning.

### Multifractal analysis

Multifractal analysis generalizes standard random-walk diffusion analysis that estimates how standard deviation grows as a Hurst exponent *H* of time^50^. Conceptually, diffusion is the spread of a particle as it flows through its surroundings, but it applies to biological sciences as a way to quantify how variability of behavior grows with time. The general notion behind null-hypothesis significance-testing is that behavior varies due to random processes even without or besides the influence of an experimental manipulation. Diffusion is the variation due to random processes, and diffusion analysis seeks to identify the random processes that give rise to observed variation.

Pearson (1905)^51^ invented the random-walk as a theoretical metaphor useful for generating different types of diffusion in terms of discrete steps taken along a continuous path: in Pearson’s original formulation, the random-walker is drunk and takes one step at a time, with each step in a direction unrelated to—or independent from—the direction of the previous step. For a one-dimensional path, the drunkard walk proceeds at each step indistinguishably from a (sober) toss of a fair coin, with the choice of one direction (50%) equally probable as the choice of the other direction (50%), and the entirety of the drunkard’s walk is an accumulation of independent deviations. The series of step numbers in between turns follows a pattern known as additive white Gaussian noise—or just “white noise.” This accumulation of steps in independent directions produces a Gaussian distribution of positions, and it is equivalent with the notion of the Gaussian distribution as a sum of very many uniformly distributed, independent random variables—with each coin toss or each choice of direction having uniform probability for each direction and with subsequent tosses/choices having no dependence on previous tosses/choices.

The shape of the position distribution and the rate at which it changes provides the rudimentary criteria for what is called “ordinary diffusion.” The mean of this Gaussian distribution of positions is zero, suggesting that the best place to look for the drunkard is right at its starting position, and the variance increases linearly with time, indicating that the standard deviation necessarily increased at the square root of time (i.e., standard deviation increases with time raised to an exponent of 0.5). Pearson’s drunkard and its standard-deviation growth with square-root time typified what became known as “ordinary diffusion.” There will of course be unsystematic error, and so the standard deviation may vary unsystematically from the square-root of time, i.e., unsystematically from the 0.5 exponent on time, but ordinary diffusion should maintain an exponent on time that is within a 95% confidence interval around 0.5. This exponent appears below as *H*, and important to note for what follows is that the values estimated for *H* are all significantly different from 0.5, i.e., well outside the 95% confidence interval covering the hypothesis from ordinary diffusion.

Deviation from the classic drunkard-walk formulation moves the random walk from producing “ordinary diffusion” to producing “anomalous diffusion”. If we formulate the random walk so that the walker chooses the same direction slightly more often than 50%, then the exponent *H* will increase beyond 0.5, with the increase in *H* directly varying with the increase beyond 50% choice of the same direction. That is, if we model a more persistent random walker, the degree of persistence directly increases *H*. Hence, we use the diffusion analysis to estimate *H* to determine the degree of persistence in the underlying behaviors composing the measured series. For instance, we expect that relatively long notes will follow relatively long notes slightly but significantly more often than relatively short notes and vice versa. Hence, our null hypothesis is minimally that bird notes follow a purely ordinary diffusive process, with equal probability of short or long notes no matter what length of preceding note.

The rest of our hypothesis is that the random-walk diffusion might follow not just one exponent *H* but several and that the random-walk diffusion specifies this variation in *H* above and beyond what is simply unsystematic noise due to sampling error. Like every statistical estimate from observed data, any estimate of *H* will necessarily be an average summary with a corresponding standard error representing unsystematic variation in the underlying structure. The classic assumption that diffusion is just the accumulation—a literal summing together—of steps follows from a deeper linear-modeling assumption that the whole process is equivalent to the sum of its constituent parts. For instance, whether or not subsequent directions are more or less dependent on previous directions boils down to the average bias of the coin tossed at each step. As typically defined for linear random-walk models, persistence is determined by the average bias of the coin, and because the bias never changes, every coin toss is an isolated event roughly equivalent to all other coin tosses, and the actual sequence is irrelevant to the cumulative distribution of positions.

Our hypothesis is, in effect, that bias of the tossed coin evolves over the course of the random walk, entailing an interdependence of current activities with previous activity at many different time scales. Hence, we expect that the birdsong notes proceed with not just one mode of persistent diffusion but several because the entire sequence acts out interactions across time scales that cause persistence to change, that is, to ebb and flow with the musical expressivity noted above, and so we expect variation in *H*, and we expect that variation to be specific to the original sequence of note lengths. The null hypothesis is that order does not matter and that persistence is always the same, and so, any variation in *H* should follow simply from a comparable average persistence, represented the average bias across all coin toss.

Random-walk diffusion analysis is a class of methods for time series that treats measured time series as the step-numbers between turns, takes the cumulative sums of these increments to approximate the positions of the random walk, and estimates how fast the standard deviation grows over progressively longer time windows. That is, random-walk diffusion analysis aims to assess standard deviation in non-overlapping time windows of various sizes to determine what the relationship is between growth of standard deviation with time. This relationship emerges as the exponent *H* on time.

Multifractal detrended fluctuation analysis brings two elegant elaborations to standard random-walk diffusion analysis. First, detrending the cumulative series of positions allows us to make more conservative estimates of diffusion. For instance, the linear trends in the cumulative position series might reflect “drift” rather than diffusion—drift being a directional change determined by local artifacts/conditions and not indicative of intrinsic properties of the diffusing particle/walker. So, detrended fluctuation redirects our interest from raw standard deviation of cumulative positions to the root-mean square (RMS) of residuals on the linear fits within time windows of cumulative positions. Crucially, RMS is the standard deviation of the linear change, and in the event of zero linear change, RMS residuals will very closely match raw standard deviation. Second, multifractal analysis generalizes the RMS residuals beyond the conventional squaring of residuals and square-rooting of the resulting mean, and it replaces the 2 in the squaring exponent (i.e., 2) and square-rooting exponent (i.e., ½) with a continuously varying *q* that allows differential emphasis of small and large residuals by *q* < 2 and *q* > 2 respectively. The linear model’s classical treatment of *q* = 2 as constant reflected the deeper assumption of homogeneity of underlying fluctuations: e.g., the steps of the random walker are always the same length, and the independence of directions entailed that step-numbers in between changes of direction are roughly the same throughout. The linear model provides no expectation that diffusion should proceed according to a different exponent *H* on time for larger or smaller fluctuations. Multifractal analysis allows us to test this assumption and to learn, from any systematic deviations from the same *H*, how diffusion might change according to any of the following: the interdependence of time scales, the heterogeneity of fluctuations, or both.

**Figure 3:**
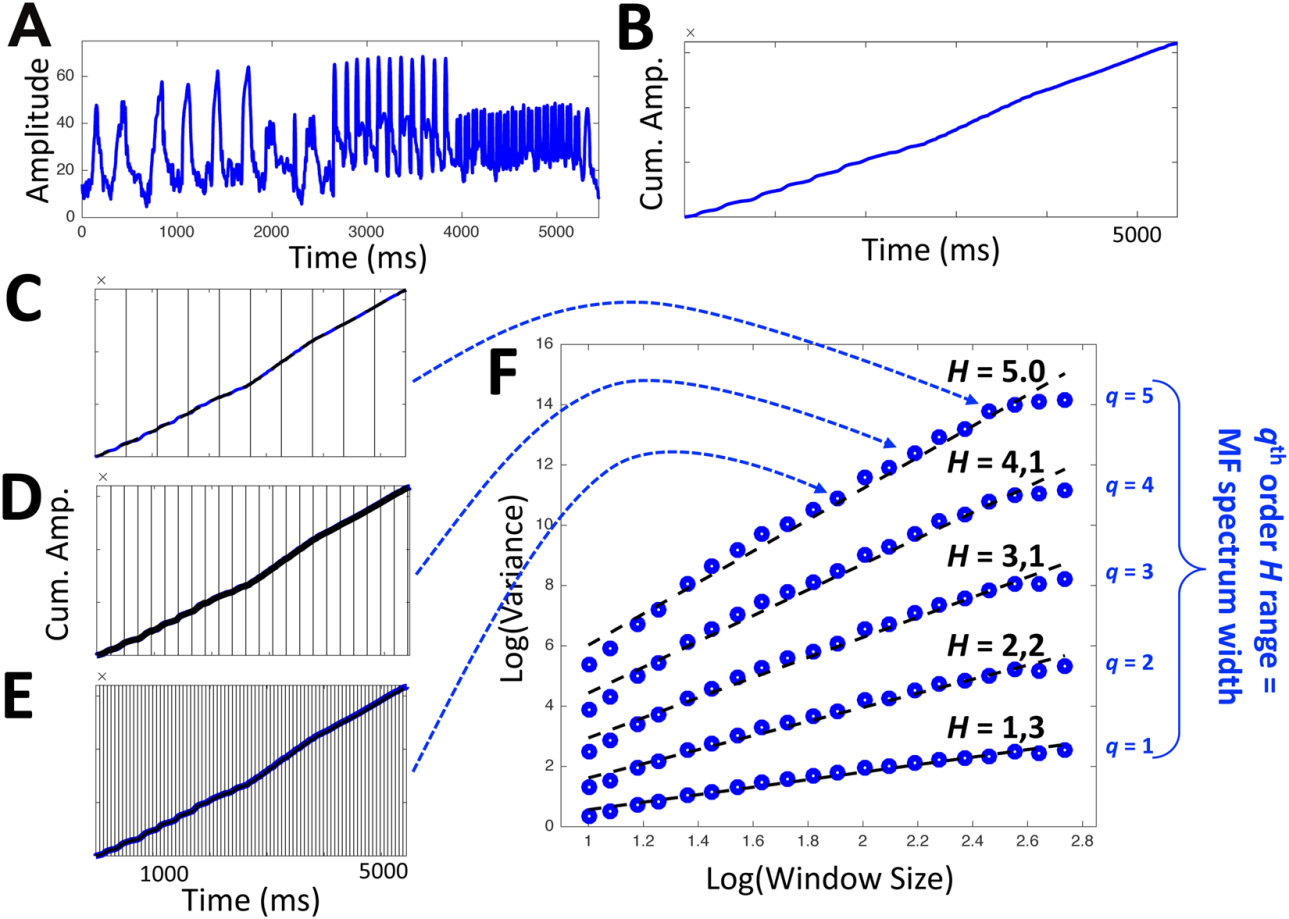
Schematic illustration of multifractal analysis. (**A**) Amplitude envelope of a song and (**B**) its integration (i.e., the cumulative amplitude over time). (**C–E**) Three different window sizes for computing *q*-order deviation for the cumulative amplitude series. (**F**) Log-log plot of average *q*-order deviations vs. window size. The more long-range structure is present in a signal, the more strongly *H* differs with *q* (i.e., the slope of the linear fit steepens with increasing *q*).

A signal like white noise, which lacks any systematic long-range structure, has a constant growth of standard deviation as an exponent *H* of time (= within successively larger time windows). In contrast, a signal that contains long-range structure (like the waveform of a song with four stanzas and a chorus) contains systematic fluctuations of variability across different time windows, and its standard deviation will therefore not grow uniformly across time scales. To estimate the development of the standard deviation across time-scales, we proceeded as follows: First, converting series *x*(*t*) (in our case, a song’s amplitude envelope) of length *N* (Fig. 3A) into a random walk-like time series *Y*(*t*) entails integration (i.e., taking cumulative sums; Fig. 3B).

1 $$Y(i)\equiv \sum _{k=1}^{i}{x}_{k}-\langle x\rangle ,i$$

The cumulative-summing step is to allow the analysis to determine the mean-square residual from linear fits in each of *N*_*s*_ non-overlapping time windows of length *s* (10 ≤ *s* ≤ *N*/4; Fig. 3C–E), where *s* corresponds to the number of data points in each window. Note: Because the length *N* of a time series is not always a multiple of the considered window sizes *s*, a portion of the data points at the end of the time series might not be incorporated into the analysis. In order to include information from those data points, the same procedure is repeated starting from the end of the time series, resulting in a total number of 2*N*_*s*_ segments. Following Eq. 2 of Kantelhardt *et al*. (2002)^50^, linear fits *y*_*v*_(*t*) of random walk-like time series *Y*(*t*) within each time windows *v* (1 ≤ *v* ≤ *N*_*s*_) leave mean-square residuals *F*^2^ for each *s*:

2 $${F}^{2}(v,s)\equiv \frac{1}{s}\sum _{i=1}^{s}\{Y[(v=1)s+i]$$

Square root of the average *F*^2^ across all time windows *v* of the same length *s* provides standard deviation *F* for each timescale *s* as in Matlab codes 4 in Ihlen’s^52^ RMS step:

3 $$F(s)\equiv {\{\frac{1}{2{N}_{s}}\sum _{v=1}^{2{N}_{s}}[{F}^{2}(v,s)]\}}^{1/2}$$

Because *H* is the exponent on time defining growth of standard deviation, diffusion analyses estimate *H* as the slope of a double-logarithmic relationship

4 $$\mathrm{log}\,F(s) \sim H\,\mathrm{log}\,s.$$

Multifractal analysis generalizes *H* with a *q*-order parameter that elaborates upon the squaring and square-rooting of standard deviation with *q*th order and *q*th-rooting, replacing Eq. 3 with Eq. 4 (Fig. 3F) from *q*_*min*_ to *q*_*max*_ (in the present study, ranging from *q*_*min*_ = 0.1 to *q*_*max*_ = 5.0):

5 $${F}_{q}(s)\equiv \{\frac{1}{{N}_{s}}{\sum _{v=1}^{{N}_{s}}[{F}^{2}(v,s)]}^{\frac{q}{2}}\},$$

for 0.5 < *q* < 5. One way to understand this application of *q* is, first, to recognize that Eq. 5 operates on the same principle of classic definitions of standard deviation. What the formula for standard deviation seeks to capture is the average distance from the mean in terms of squared distance but without leaving that average warped by nonlinearities incurred by squaring individual differences from the mean. The formula squares difference scores in order to get an average difference score that is not zero, but then it applies the inverse of squaring to that average. That is, RMS calculation is an attempt to weight the individual contribution of individual difference scores but to prevent individual-contribution weighting from exaggerating the standard-deviation measure needed to represent the entire sample. The only shortcoming is that this calculation’s constant value of *q* = 2 provides the most effective measure of variability only under the assumption of homogeneity of fluctuations. Values of *q* greater than 2 will give greater weight to larger residuals than to smaller residuals, and values of *q* less than 2 will give greater weight to larger residuals. In all cases, the q-th rooting by the 1/*q* exponent merely assures that the resulting mean residual measure will be sufficiently standardized to warrant comparison of residuals *F* for all values of *q*.

The application of continuously varying values of q is, at worst, harmless and, at best, crucially for revealing different diffusive modes within the same time series. Certainly, if the fluctuations in a time series are in fact homogeneous, the application of continuously varying values of *q* as in Eq. 5 will not yield different results. So, if the time series really is diffusion made up of homogeneous fluctuations, then there is no chance that generalizing *q* can provide false evidence of nontraditional diffusive patterns. However, the payoff of this *q*-generalization is that it will reveal different diffusive patterns in the event that a time series does draw its variability from the fact of heterogeneous fluctuations. On the other hand, failing to generalize *q* and so only applying the monofractal variant of detrended fluctuation analysis will risk the possibility that a time series’s underlying heterogeneity will go undiagnosed, and this point is precisely what previous research has intended by showing that a singly estimated *H* is by itself extremely ambiguous^53^.

If multifractal detrended fluctuation analysis shows a variety of *H*, then it indicates that the observed diffusion depends on an underlying distribution of heterogeneous fluctuations. In the event of a variety of *H*, then we revisit the aforementioned potential that observed diffusion follows from the interdependence of diffusive activity across multiple time scales, that is, from the development of bias in the random-walk choices of direction. A single MF-DFA outcome cannot answer this question alone but requires comparison of several MF-DFA estimates for a population of surrogate time series representing the null hypothesis of no interdependence, namely, that diffusion would proceed according to independent time scales in which the bias in choices of direction remains the same throughout.

### Surrogate analysis

Spurious differences in *H* across different *q* can result from linear autocorrelation or distribution properties, without being indicative of any long-range fluctuations in variance. Hence, multifractality of the original signals are commonly compared to surrogates that preserve linear properties (i.e. distribution, linear autocorrelation) of the original signal, but lack all nonlinear structure. We compare the range of *q*th-order *H* (multifractal spectrum width) for each original with 100 surrogate amplitude envelopes generated with the Iterative Amplitude Adjusted Fourier Transform (IAAFT)^54^. IAAFT begins with a Fourier transform to estimate the amplitude and phase spectra. Subsequent iterations randomize the phase spectrum, applying inverse-Fourier transformation to combine it with the original amplitude spectrum, and then replace rank-ordered values of this new series with those of the original series.

Evidence for systematic variance fluctuations/long-range structure across hierarchically nested timescales is a multifractal spectrum width for the signal in question that lies beyond the 95% confidence interval its IAAFT surrogates^48^.

### Testing synthetic “average-rhythm” songs

We classified note types through manual annotation, using visual and auditory inspection with Sound Analysis Pro 2011^55^ and GoldWave v6.18. For each type, we created an envelope profile by averaging note duration and intensity course across all instances. Pause types (as identified by the two adjacent note types) were averaged for duration (pause amplitude being set to zero). Using these averaged note and pause profiles, we generated synthetic songs using the original sequential arrangement. Multifractal spectrum widths of these “average-rhythm” songs were then compared to their IAAFTs like the original songs.

To this end, we used a paired-sample *t*-test comparing the distances of the original songs to their IAAFTs and the average-rhythm songs to their IAAFTs (in standard deviation [*SD*]).

## Results

**Figure 4:**
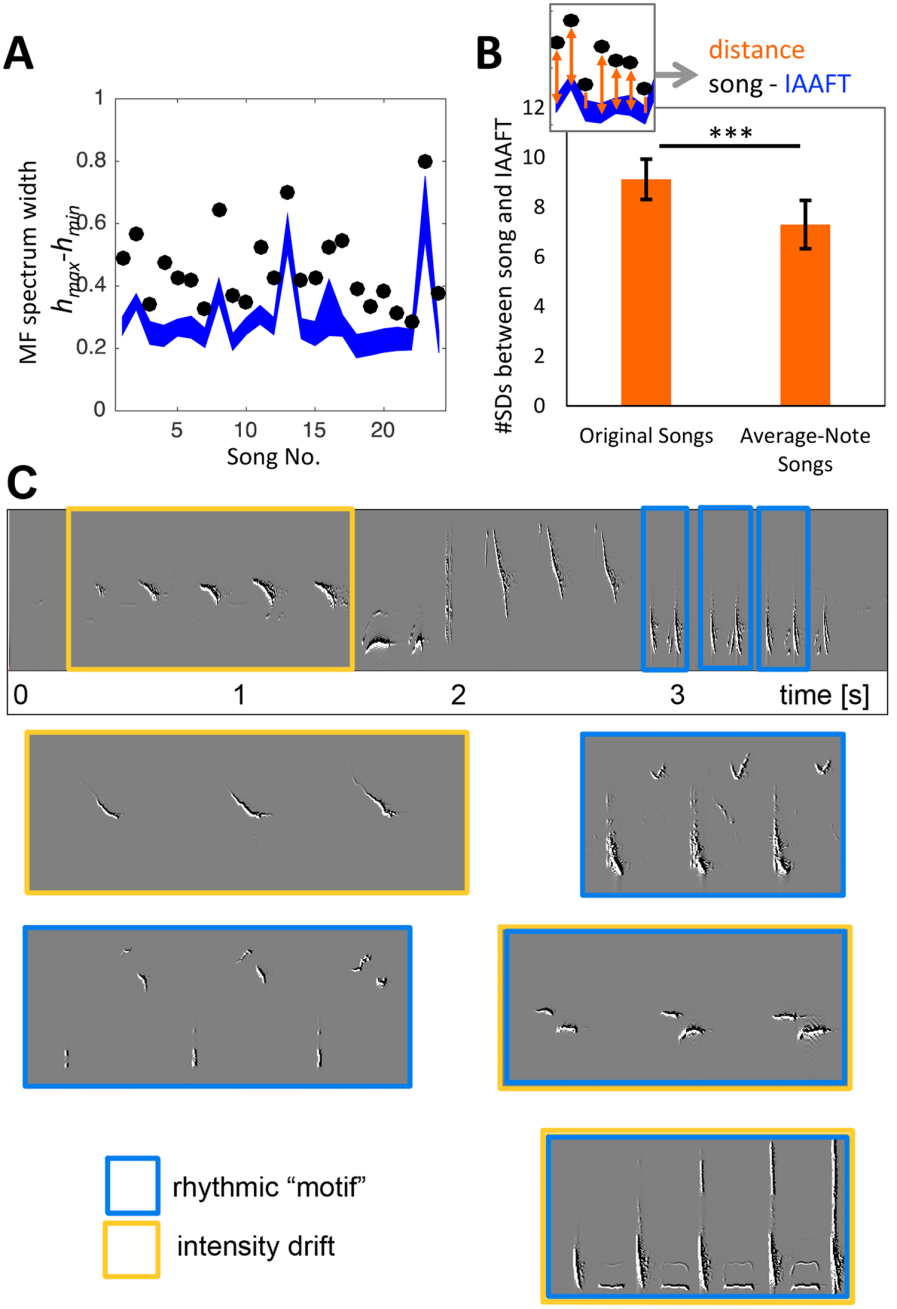
Thrush nightingale rhythms are multifractal, due to both sequential arrangement and timing deviations. (**A**) Multifractal spectra of the 24 original songs (black circles) are wider than expected from their IAAFT surrogates (blue). Blue area = 95% confidence interval of the surrogates. (**B**) Multifractality in original rhythms is significantly greater compared to rhythms of “average-rhythm” songs. Bars represent *difference* between amplitude envelopes and their IAAFTs (inset), measured in *standard deviations from the mean* of the IAAFTs’ multifractality values (*h*_*max*_ − *h*_*min*_). Averaging note amplitude envelopes significantly reduced multifractality (*p* < 0.001). (**C**) An entire thrush nightingale song containing examples of an intensity drift (yellow) and a rhythmic motif (blue), and five more example subphrases from different songs.

To identify any systematic long-range variability fluctuations in the thrush nightingale rhythms, we first calculated multifractality of the original songs’ amplitude envelopes, and compared this value to the multifractality value of their IAAFT surrogates (control time series with the same distribution and linear autocorrelation properties but lacking any long-range correlations; see Methods). All 24 songs’ amplitude envelopes exhibited multifractality, i.e. non-zero ranges in the *q*th-order *H* (Fig. 4A). Further, multifractal spectrum widths for original amplitude envelopes (black circles) are above the 95%-confidence intervals of their corresponding IAAFT surrogates. This indicates that song amplitude envelopes exhibit non-local changes in variability, systematically going through more and less variable phases across different timescales.

We next compared the original rhythms to the average rhythms of songs that we stripped off any subtle timing and amplitude deviations, to test whether birds may add expressiveness to their vocal sequences by adding systematic timing/intensity fluctuations. The average-rhythm songs turned out still more multifractal than their IAAFT controls, but less so than the original songs: A paired-sample *t*-test comparing the distances of the original songs to their IAAFTs and the average-rhythm songs to their IAAFTs (in *SD*) revealed that the originals are significantly more multifractal compared to their matched average-rhythm songs (Fig. 4B; *t*(23) = 2.92, *p* = 0.008). That is, the original songs differ more strongly from their IAAFTs than the average-rhythm songs. Note, however, that some of the distributions of note and pause durations are skewed. Hence, averaging pauses might not be reliable for some durations. However, if median durations are used instead of average durations, the same effect pattern is observed (*t*(23) = 5.66, *p* < 0.001).

**Figure 5:**
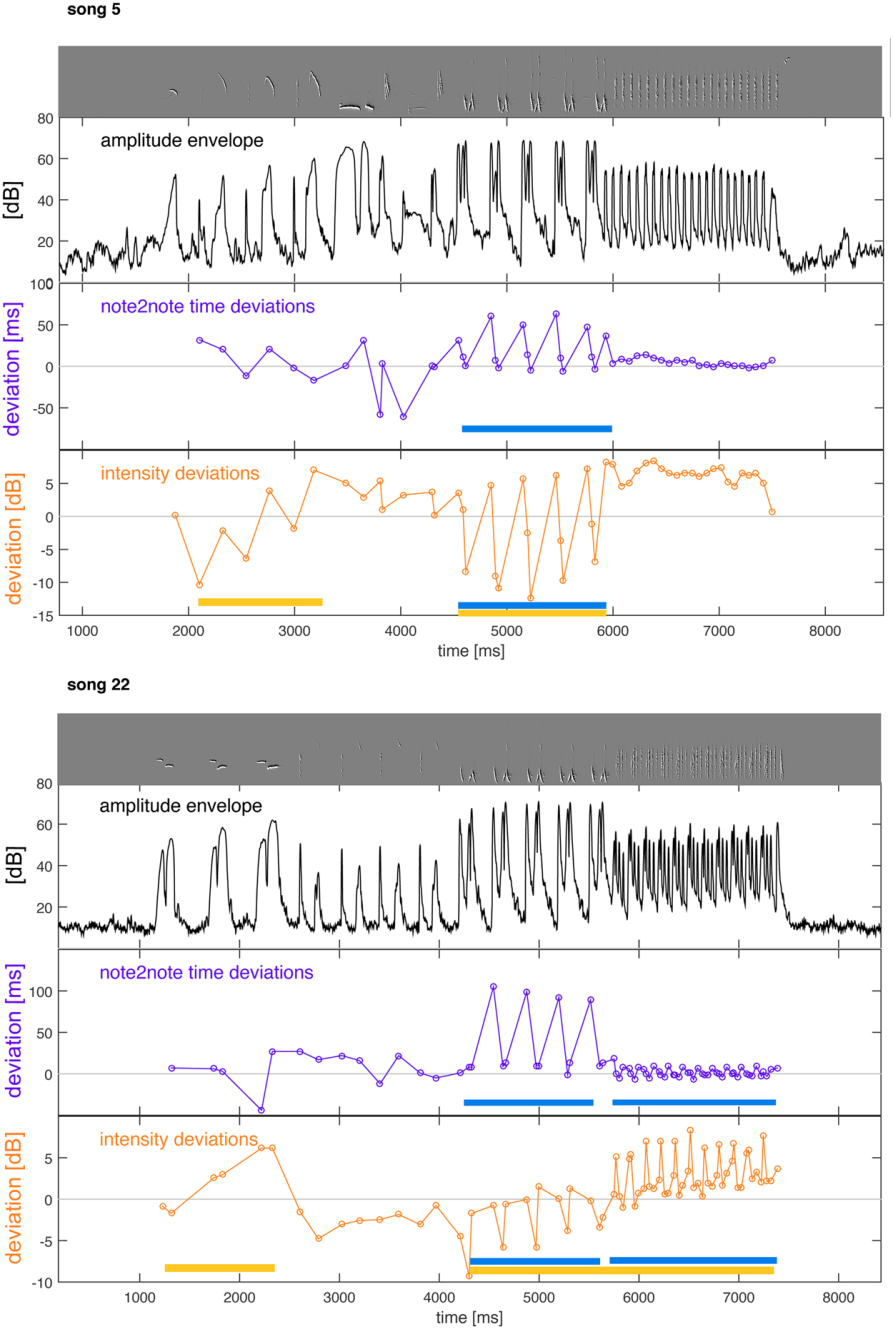
Course of timing and intensity deviations across two example songs. Upper panels of each song (gray) are sonograms, second panel (black) shows the amplitude envelope, third panels the deviations in note onset-to-onset time (purple) from the average onset-onset time of the respective note transitions, and bottom panels the deviations in note intensity (orange) from average intensity of the respective note type. The deviations contain both phases of drift (indicated by yellow bars) and recurrent deviation patterns (blue bars), sometimes both at the same time.

Thus, a significant part of the original rhythms’ multifractality originates in non-random deviations from average note/pause profiles. Another part of the multifractality – the part still present in the average-rhythm songs – is due to the sequential arrangement of notes. Drifts and motif recurrences that contributed to the rhythms’ multifractality can be apparent in the sonograms (Fig. 4C). Visualizing examples of the course of the within-song *deviations* from average notes in timing and intensity (Fig. 5) lends plausibility to a scenario in which deviations induce both drifts and recurrences: Both timing and intensity deviations develop in clearly non-random, structured ways in the two song examples in Fig. 5, in which periods of drifts alternate and mix with repeating “motifs” of deviation patterns. Note that such repeating rhythmic “motifs” (marked in blue in Figs. 4C and 5) bear structural resemblance to “swinging” rhythms in jazz music that feature regular long-short patterns^43^. Both drifts and recurrence patterns are common and occurred in almost every song, often (but not always) overlapping. The portion of the song covered by these patterns typically lies in the range of 20–70% for drifts, and 40–90% for rhythmic motifs.

## Discussion

Using multifractal analysis, we show that the rhythms sung by a thrush nightingale contain long-range correlations across multiple timescales, and that part of this structure is due to subtle timing and intensity deviations from average note/pause profiles: Eliminating the subtle deviations resulted in significantly reduced multifractality, indicating that these deviations are not random. Instead, they contribute to systematic long-range correlations in the songs’ rhythm – for instance, by adding drifts or recurrent rhythm patterns to the note sequence. However, songs stripped off all these subtle deviations were still significantly more multifractal than their IAAFT controls. This indicates that another part of the rhythms’ multifractality is due to sequential arrangement of the particular note types: By combining notes with specific internal structures into sequences, the birds also generate long-range rhythm patterns, which may materialize as drifts or recurrences. Note that additional *random* noise – for instance originating in noisy neuronal signals or imprecise muscle control – does not add to multifractality (which precisely indicates *non-random* fluctuation patterns).

Our results are the first to reveal multifractality in birdsong, and show that multifractal analysis can be successfully used to determine the extent of structuredness in animal vocalizations.

While our analysis does not reveal to what extent drifts and recurrent timing patterns, respectively, contributed to the observed multifractality, some instances of either are apparent to the eye both in the sonograms (Fig. 4C) and in the course of the intensity and timing *deviations* within songs (Fig. 5). This suggests that such patterns are indeed one source of the measured multifractality (possibly among others).

In sum, our results are consistent with the hypothesis that songbirds and musicians use similar techniques of combining expected and unexpected patterns to attract their listeners’ attention. Two mechanisms to do so are (1) arranging notes and (2) adding expressive timing to a song in a way that long-range rhythm structure emerges, for example as recurrent timing/intensity patterns or drifts. Systematically testing this hypothesis will require further experimental research to assess whether birds can perceive such subtle, multifractality-increasing timing structure^56^, whether they actually prefer it over “average” note timing, and whether these patterns are indeed under active control (evidence for this could be that they are *learned*, reflect experience, or differ between social contexts). Until shown that these multifractal timing patterns are indeed actively produced and more efficient in attracting and/or holding the attention of avian listeners, however, other plausible explanations (some of them more trivial) are available, and will be discussed further below.

### Functional relevance of multifractal timing structures

At first sight, our observations and interpretation might seem at odds with the well-documented (albeit not unchallenged^57–59^) finding that in many bird species, conspecific listeners appear to prefer vocal consistency and stereotypy over variability in song (for a review, see Sakata & Vehrencamp^60^): For instance, when producing undirected song (i.e. singing by themselves), male zebra finches sing in more variable, less stereotyped ways as compared to song directed towards a female, and female zebra finches prefer the latter variant^61^. A closer look at the notion of “vocal consistency” shows, however, that our data do not contradict these findings, and moreover, preferences across species are more diverse and complex as to be fully captured by a general preference for “similarity across renditions”.

First, although our observed timing deviations from average sequential note timing will increase variability in some measures, like intensity course across instances of a note, they do not clearly indicate lower overall song consistency. The deviations do not add *random noise* to the signal. On the contrary: the fact that they increase multifractality precisely suggests that they further *structure* the signal by creating systematic long-range patterns, and that this additional structure shows systematic correlations across different time scales. In essence, the notion of consistency (vs. variability) highly depends on the domain or level it is applied to: Long-range timing structure may decrease single-note instance consistency, but could at the same time increase consistency on other levels, like between song instances.

Second, a closer look at what kinds of consistency are reportedly preferred by avian listeners suggests that these are quite different from our multifractal timing patterns. Our study measures note timing and intensity *within* songs, whereas none of the studies on listener preference is based on such rhythmic features. Most direct evidence for listener-preferred consistency (as measured by reproductive success or behavioral measures like copulation solicitation display, approaching behavior, or singing response) has been reported for *spectral* features of notes, often in combination with note rate, when trill performance was concerned^62–67^, not for within-song rhythmic timing.

In addition, different studies failed to detect a preference for consistency: Spectral note consistency is not preferred by house wrens (*Troglodytes aedon*)^68,69^, and consistency in within-motif syllable *sequence* does not explain variance of female preference in zebra finches (*Taeniopygia guttata*)^70^. Together, these findings suggest that where occurring, the preference for consistency is likely both species-specific and feature-specific.

We do not know whether thrush nightingales prefer consistent *spectral* note features. But if they do, this preference does not have to translate to the time domain: A preference for long-range timing drifts and recurrences could occur independent of spectral preferences. Interestingly, this seems to be the case in (at least Western) instrumental music: While in the time domain, drifts and recurrences are a common musical pattern and often coded in the scores, pitch drifts (beyond *vibrato* within a note) are quickly considered incorrect, and impossible to perform on many (e.g. keyboard) instruments. Timing drifts in music are appreciated by human listeners, as machine-generated music featuring “exact” timing sounds less attractive to us than music containing timing inaccuracies, especially when the latter contain long-range correlations instead of being random^45–47^. Experimental studies in thrush nightingales are needed to test whether listeners actually prefer multifractal variation over both random variation and complete consistency.

Species might differ considerably in what song features they prefer to be more consistent, or more varied. While this aspect of birdsong has not received much scientific attention^71^, it might be an important source of the stark structural differences between different species’ songs: While, for instance, zebra finches show most variety on the level of bout structure (vs. little or no variation within their motifs or notes^72^), species with large repertoires like the European nightingale (*Luscinia megarhynchos*) show an extreme variety on the level of song types, but little variety within “bouts” (which are the relatively stereotyped instantiations of the many song types from their large repertoire)^73,74^. Interestingly, in pied butcherbirds (*Cracticus nigrogularis*), individual song is characterized by a tradeoff between repertoire size and sequential variability: Individuals with larger motif repertoires produce these with strong sequential regularity, whereas individuals with small repertoires deliver them in a more irregular way^25^. In each of these examples, species-specific song structure includes both variable and stereotyped parts, they are just represented on different, species-specific domains or levels of the song.

What could be the function of such a structure in a communication signal? We think that an appealing and parsimonious explanation lies in the proximate ecological goal of birdsong: to eventually alter a conspecific listener’s behavior. To this end, birdsong needs to be effective in attracting and holding the listener’s attention^24,25^. To prevent habituation in the listener^75^, the song must be *varied* in some way (since a completely uniform, consistent repetition of a single element, even if it was a particularly “sexy” syllable^76^, would lead to quick habituation – which might be the reason birdsong rarely, if ever, sounds like this). On the other hand, the signal should not fluctuate *randomly*, as such a signal might also induce habituation quickly: despite maximized variation, the listener’s parsing of such a signal would be a flat, uniform process in which memory and expectation play no role. A signal in which varied and stereotyped structures are balanced – in other words, a signal with multifractal properties – might just be the most efficient way to invite conspecific birds to listen. Music psychology has suggested that this is the way music “works”^26,27,77^, and birdsong might, too^24,25^.

Of course, alternative (or additional) explanations are available for the timing pattern properties observed here. Signals characterized by both variability and stereotypy may be effective at *encoding* certain kinds of information – for instance about both the species affiliation and individuality of the singer at the same time^78–80^: In a learned communication system like birdsong, variable features might be differentially used by individual birds, making them identifiable by their song, while at the same time, species-specific, stereotyped song features can serve for species identification. A particular functional significance of more varied song aspects could lie in a greater potential to change^81^, for instance with experience^82–84^: If individual birds sharpen their multifractal timing patterns throughout life, these could serve to signal the singer’s singing experience, as a proxy for life experience. A driving force particularly promoting stereotypy, on the other hand, might be memory and attention capacity: In both singers and listeners, memory and attention span are limited^85^, and learning, producing and evaluating^26,86,87^ songs are probably facilitated by a more stereotypical temporal patterning of the songs.

Finally, acoustic features of birdsong have often been suggested to function as “honest signals”, directly indicating a superior quality of the singer by being harder to produce than other song features, and in numerous cases particular song features have indeed been found to significantly correlate with measures of singer quality^71,88^. However, complex multifractal timing/intensity structures do not impose themselves as compelling honest signals: We cannot think of any reason why these patterns would be particularly hard to learn or produce, or how else they would be indicative of any other qualities than singing itself. Luckily, they might not have to be: Picking up Darwin’s ideas on sexual selection, Richard Prum has recently outlined a theory of “aesthetic evolution” that can account for the evolution of aesthetic stimuli (like ornaments, song, or dance) in the complete absence of honest signaling, simply by virtue of a co-evolutionary process in which a trait evolves together with its sensory evaluation^89–91^. This process is driven by assortative mating between individuals carrying the trait and individuals preferring it. The trait thus functions as a display, and this display and the mating preference for it shape each other over evolutionary time – not because the display indicates quality, but simply because it functions through the perception and evaluation of other individuals and affects mate choice. This leads to a cumulative effect of many individual mating decisions on the shape of the display; the species thus functions as a selective force in its own evolution. This mechanism offers a “null hypothesis” for the evolution of displays and their preferences – an attractive model especially in the case of a display trait that does not plausibly indicate quality. Note that this model does by no means assume displays *never* function as honest signals. But it offers a general and mechanistic account for the evolution of displays and preferences through sexual selection in the absence of additional selection on either the display trait or the preference. Applied to our case, the multifractal timing patterns observed in the song might in fact be preferred by listeners (which stands yet to be tested empirically!) for no other reason than that they sound nice to them.

### Implications of different timing structures for possible generative processes

Interestingly, the two patterns – drifts and recurrences – and their origin in note arrangement and timing deviations, respectively – have different implications for the neural mechanisms underlying song timing.

We assume that note sequences are generated by a central note sequence generator that arranges note types into sequences. The *deviation-based rhythmic structure* we identified in the songs (Fig. 3B) points to some additional effect on timing that operates on top of (or in parallel to) the sequence generator. In the case of deviation-based *drifts* (as in Fig. 2, right, middle panel), this additional effect may simply consist in entirely involuntary processes, like peripheral constraints (e.g. slowing down due to muscle fatigue^92^), or fast attention or arousal fluctuations. While such sources may better account for slowing and quieting as opposed to speeding up or rising (as occur in the examples in Fig. 5), we certainly cannot infer from deviation-based drifts the presence of a centrally controlled mechanism governing note timing. However, *recurring patterns* (as in Fig. 2, right, lower panel) seem harder to explain by peripheral or other mechanisms that are not under the control of the bird. These patterns might indeed be indicative of centrally controlled generation mechanisms. Whether there is a mechanism explicitly dedicated to controlling note timing or rhythm, or rather one that affects timing trough governing other processes (like, for instance, hierarchical arrangement of note subphrases, where within-subphrase note timing follows other timing dynamics than between-phrase timing) will be an interesting question for future research.

As far as we can see, a centrally controlled generative mechanism is also required for any *syntax-based rhythmic structure* (Fig. 2, left). We assume that arranging different note types in ways that give rise to systematic long-range timing patterns requires

- a neural representation of the discrete identity *and* internal features of the note types sung (i.e. their duration, intensity, amplitude course).
- These note feature representations must be accessible to a central timing-affecting mechanism that operates on the time-scale of (sub-)phrases to create the resulting patterns.

Note that this is in contrast to a situation in which note timing would simply result from gesture transition, plus attention drift and peripheral constraints. We cannot think of any way in which such a mechanism alone would be able to generate *syntax-based* rhythmic structure as described in Fig. 2 (left). Interestingly, syntax-based long-range timing structure is also incompatible with the simple Markovian models for syntax generation that are widely accepted for many other bird species’ songs: In a Markov chain, the probability of a given element to occur in a position depends on only a limited number of preceding elements. While a first-order Markov model of sequence generation may still be a plausible syntax generator^21,22,93^ for sequences whose long-range structure originates in subtle deviations only (Fig. 2, right), it cannot account for sequences with long-range structure due to sequential arrangement (Fig. 2, left).

In sum, the multifractality we observe in the average-rhythm songs whose timing depends only on note arrangement is consistent with thrush nightingale note timing being under the control of the birds, their rhythm being more complex than what a simple Markovian model of sequence generation could specify, and with a generation process as described under (a) and (b). However, further research is needed to provide conclusive evidence: First, analyzing more individuals will determine how general a phenomenon amplitude envelope multifractality is, and whether individuals differ with respect to it. Second, while multifractal analysis can globally quantify multi-timescale structuredness of a time series, it does not specify which structures in particular are contributing to the complex signal’s multifractality. Further studies should determine the exact roles of drifts vs. recurrence patterns in song multifractality, and how exactly they result from syntax-based vs. deviation-based timing patterns. Third, comparative studies including more bird species are needed to investigate whether such multifractal timing patterns are common to all birdsong, or a property specific to certain species only.

### Similarity to musical rhythm and to speech prosody

So far, we have stressed how our results underline similarities between birdsong and music: We pointed out how long-range correlations similar to the ones we found in the thrush nightingale rhythms have also been described for musical rhythm, how multifractal timing deviations in music have been shown to be recognized by listeners and preferred over “exact” rhythm^46,47,94^, and we hypothesized that the multifractality measured here reflects expressiveness intentionally added by birds and humans to render song more attractive (a hypothesis that invites further research to test whether multifractal vs. non-multifractal rhythm patterns are rated more attractive by thrush nightingale listeners). The comparison between musical and avian rhythm is crucially motivated by their common proximate function of engaging the minds of listeners, and holding their attention.

However, another interesting *structural* parallel exists with speech prosody (sentence melody)^95^: in both prosody and the multifractal patterns we investigated here, intensity and timing fluctuate in systematic ways on suprasegmental scales exceeding the terminal elements, notes and words^96^. The notion of speech prosody additionally includes pitch fluctuations, a feature not covered by our analysis in birdson (where it is perhaps more akin to categorical musical pitch than to the relative pitch shifts in prosody), but similarities in structural organization of prosody and birdsong rhythm fluctuations might nevertheless run deep: Speech prosody is interconnected with (but not determined by) semantics and syntax, with boundaries that are often, but not always, coinciding, and with a hierarchical organization whose levels are characterized by specific prosodic structures (“prosodic hierarchy”). Finding within-song timing and intensity fluctuations to be multifractal may point to a similar hierarchical organization which multifractality has indeed been interpreted to reflect^49^. Comparing the course of deviations to the amplitude envelopes (Fig. 5) suggests that boundaries in syntax and in the intensity/timing fluctuation patterns may often coincide as well, perhaps indicating a similar interconnectedness between syntactic and timing/intensity structure.

Prosody in speech contributes to various functions, such as coding the form of an utterance (question vs. command or statement), signaling emotional state of the speaker, or directing the semantic focus. Semantic and propositional aspects are not applicable to birdsong, but signaling of emotional state could be a function common to both^95^.

It is conceivable that parallels between speech prosody and the timing/intensity structures in the thrush nightingales songs extend to aspects of their neural control: A timing-affecting mechanism with the above-mentioned properties a) and b) might be analogous to the processing streams in the human brain assumed to process prosody, which are separate from and working in parallel to the processing streams that underlie core linguistic abilities such as phonology, syntax, and semantics^96^. Interestingly, besides their structural parallels, the *perception* of speech prosody is correlated with musical rhythm perception in human listeners^97^ and might rely on shared neural mechanisms^98,99^ – underlining that parallels to either may be useful to inform birdsong research.

### Data accessibility

Data is accessible from the xeno-canto birdsong library (for details, see Methods 2.1).

## Electronic supplementary material

Supplementary Information
