# Supplementary material for: Multifractal analysis reveals music-like dynamic structure in songbird rhythms
Source: Sci Rep. 2018 Mar 15;8:4570. doi: 10.1038/s41598-018-22933-2 (PMC5854712; doi:10.1038/s41598-018-22933-2)
Supplement: Supplementary file 1 — Supplementary Information [file 41598_2018_22933_MOESM1_ESM.docx]

**Supplementary Material**

to the manuscript

**Multifractal analysis reveals music-like dynamic structure
in songbird rhythms.**

Authors: Tina C. Roeske^1^*, Damian Kelty-Stephen^2^, Sebastian Wallot^1^

^1^Max Planck Institute for Empirical Aesthetics, 60322 Frankfurt, Germany

^2^Department of Psychology, Grinnell College, Grinnell, 50112 IA, USA

* corresponding author (christina.roeske@gmail.com)

**Supplementary figure 1: Catalog of note types corresponding to our manual annotation.** Sonograms show examples for each note type. We annotated each note type with an (arbitrary) number (underneath sonograms).
